# Type 1 and Type 2 Diabetes Mellitus: Commonalities, Differences and the Importance of Exercise and Nutrition

**DOI:** 10.3390/nu15194279

**Published:** 2023-10-07

**Authors:** Maurício Krause, Giuseppe De Vito

**Affiliations:** 1 Laboratório de Inflamação, Metabolismo e Exercício (LAPIMEX) e Laboratório de Fisiologia Celular, Departamento de Fisiologia, Instituto de Ciências Básicas da Saúde, Universidade Federal do Rio Grande do Sul (UFRGS), Porto Alegre 90035-003, RS, Brazil; 2 Neuromuscular Physiology Laboratory, Department of Biomedical Sciences, University of Padova, 35131 Padova, Italy; giuseppe.devito@unipd.it

Diabetes mellitus represents a group of physiological dysfunctions characterized by hyperglycaemia resulting directly from insulin resistance (in the case of type 2 diabetes mellitus—T2DM), inadequate insulin secretion/production, or excessive glucagon secretion (in type 1 diabetes mellitus—T1DM) [1]. Type 1 diabetes is a chronically progressive autoimmune disease that affects approximately 1% of the population in the developed world [2]. This adverse immune response is induced and promoted by the interaction of both genetic and environmental factors [3]. In contrast, in type 2 diabetes, insulin resistance coupled with reduced insulin output appears to be the major cause of hyperglycaemia (affecting approximately 8.5% of the adult population) [2].

Although the aetiology of diabetes may differ from T1DM to T2DM, common features may occur during the progression of the disease. In the case of T2DM (insulin resistance), pancreatic β-cell failure may occur in the long term, while in T1DM (pancreatic β-cell death/insulin deficiency) insulin resistance can be induced as the condition progresses [4]. Thus, similarly to both types of diabetes, particularly in the long term, insulin resistance and β-cell dysfunction/death may be present, impairing several tissues and cell function and metabolism. 

Hyperglycaemia, dyslipidaemia, and low-grade inflammation (consisting of circulating inflammatory cytokines or adipokines released by adipocyte expansion [5], and also by gut microbiota dysbiosis [6]) are considered important factors in the progression of T2DM and are generally present in obese individuals who are at risk of T2DM [7]. These conditions lead to β-cell stress and insulin resistance (through a variety of processes that mainly include uncontrolled generation of reactive oxygen and nitrogen species (ROS/RNS) and cytokine-dependent signals) [7].

Insulin resistance is also prominent in patients with T1DM and involves hepatic, muscle, and adipose tissues [8]. Weight gain caused by the administration of exogenous insulin, together with the adoption of a sedentary lifestyle (particularly related to a fear of exercise-induced hypoglycaemia [9]) and a high-calorie diet [10,11], leads to changes in an individual’s body composition, lipid profile, and blood pressure, similar to those observed in metabolic syndrome, in obesity, and in people with T2DM, generating insulin resistance and increased risk of cardiovascular disease [12,13]. The presence of metabolic syndrome in patients with TDM1 results in the phenotype called “double diabetes” [14].

Individuals with T1DM and T2DM share several cardiometabolic complications, such as endothelial dysfunction [15], changes in glomerular filtration/kidney function [16], low-grade inflammation [17], oxidative stress [17], blood coagulation [18], mitochondrial dysfunction [19], cardiac dysfunction [20,21], anabolic resistance [22], metabolic inflexibility [23], and gut microbiota dysbiosis [24]. The treatment of these conditions and the management of glucose balance may require pharmacological [25], surgical (in obese related-diabetes) [26], or, particularly, lifestyle-related interventions, such as exercise and nutritional changes [27].

Exercise is the most effective non-pharmacological tool to prevent and treat cardiometabolic diseases related to both types of diabetes [28]. As recently demonstrated, the combination of acute resistance and aerobic exercise can improve glycaemia control, metabolism, oxidative stress, inflammation, and skeletal muscle anabolic adaptations in people with T1DM [29]. Despite the negative adaptations caused by T1DM in the skeletal muscle (e.g., mitochondrial dysfunction, inflammation, and regeneration), exercise training can decrease glucose fluctuations and the occurrence of hypoglycaemia and improve skeletal muscle dysfunction [30,31]. Similarly, people with T2DM also improve their metabolic and molecular profile in response to exercise training [17,32,33,34].

The combination of exercise with dietary interventions has also been extensively studied and found to result in additional positive cardiometabolic adaptations [33,35]. The potential nutritional strategies to improve cardiometabolic health in diabetic people include protein supplementation [36], amino acids [37], probiotics/symbiotics [6], nitric oxide/nitrate donors [38,39], heat-shock response activators [40], antioxidants [41], polyunsaturated fatty acids (omega 3/6) [42], and vitamins [43].

The first studies published in this Special Issue provide new evidence of the importance of exercise and dietary interventions for diabetes [44,45]. The study by Muntis and colleagues explored the effects of protein intake on glycemia following moderate-to-vigorous physical activity in adolescents with T1DM [44]. Based on their results, the authors suggested that elevated daily protein intake may improve post-exercise glycaemic responses.

Using an animal model of obesity-induced diabetes (Western diet), D’Haese and colleagues studied the effects of moderate-intensity training (MIT) and high-intensity interval training (HIIT) on adverse cardiac remodelling and dysfunction [45]. The authors demonstrated that both intensities lowered insulin resistance and blood glucose levels compared to sedentary animals. Particularly, in the heart, MIT and HIIT lowered end-diastolic pressure, left ventricular wall thickness, and interstitial collagen deposition. In addition, positive improvements in mitochondria dysfunction were found.

The studies featured in this Special Issue provide new evidence and knowledge of how dietary and exercise interventions can improve diabetes and its associated diseases. The underlying mechanisms of the positive metabolic adaptations induced by different types of exercise and nutritional supplementation in diabetes continue to be elucidated. New research in this field is mandatory to assist in the formation of additional evidence-based exercise/dietary guidelines for people living with diabetes.
